# W. W. H. Thackston's Letter on the Turnkey

**Published:** 1841

**Authors:** W. W. H. Thackston

**Affiliations:** Farmville


Messrs. Editors—

It is with some reluctance that I avail myself of the opportunity, through your excellent periodical, of discussing a subject which merits an abler pen and more experienced mind, than it is my good fortune to possess. And permit me here to remark, that I have, from these considerations, been constrained to await, silently, the issue of several numbers of this journal, hoping in each successive one, to find the subject in the hands of some individual, whose scientific attainments, sage experience, and professional acumen, would ensure to him a more favourable reception than one so humble as myself could anticipate. The subject alluded to, is Mr. Shepherd's communication upon the Turn-Key. And here I would observe, that Mr. Shepherd's introductory remarks breathe a spirit which every high minded member of the dental profession should cultivate: and which, if cherished by its members generally, would result in consequences alike advantageous to it, and beneficial to mankind. But with regard to the opinions of Mr. Shepherd relative to the superiority of the tooth-key and the propriety of perpetuating the use of this powerful but most destructive instrument, (destructive, not only to the comfort and well being of those who may unfortunately become the subjects of its painful mutilations, but destructive to the interest of the profession.) That they are erroneous, and the positions taken to sustain them untenable, is a fact, which I shall attempt to demonstrate. And in executing this design, I shall examine first, the construction and adaptation of the tooth-key to the purposes for which it is used; secondly, the superiority of properly constructed forceps over the key; and thirdly, and lastly—the propriety of abandoning entirely, a confessedly dangerous instrument, when its place may be supplied by one of generally acknowledged superiority, by an instrument possessing all its desirable qualities, and clear of the insuperable objections which attach to it. As Mr. Shepherd truly observes, much the larger number of keys now in use are grossly disproportioned—the fulcrum being too long. And it is a fact equally true, that, however just the proportions may be—however well adapted the hook may be to the fulcrum—or whatever shape the latter may possess—the only attainable object in its application is, a powerful rotary motion; and this only by exposing the gums and alveoli to greater or less injury as the circumstances of the case may be. If the shape and situations of the fangs be favourable, that is, if they be straight, and if the circumference of the neck of the tooth be equal or greater than that of the fangs at their extremities, extraction may evidently be effected, without serious injury or inconvenience to the patient. But unfortunately for the advocates of this instrument, those are things without the range of their vision; and it but too often remains (when too late to avoid or repair the injury) for the direful consequences of an ineffectual operation, or dangerous fracture, to teach the operator—what? that the key, though an excellent one in many instances, was not adapted to the *particular* case. The frequency of those particular occurrences, I shall leave to be determined by those in the habit of generally using the instrument. But we are here met by being told that wherever the fangs diverge too much to render their removal with the key safe, that we may first give it a trial, and when such indications occur as to render its continued application imprudent and dangerous, that we must then have recourse to the forceps. And I would here ask, and ask emphatically, what are those indications, and when do they develope themselves? It is after we have exposed those who have committed themselves to our care, and confided in our skill, to the most imminent danger: when some severe contusion, or violent concussion discloses the extent of that unnecessary injury, which no man of *proper feelings* would inflict. And we are then to have recourse to the forceps. Strange logic, indeed! Use an inferior instrument to effect what its superior cannot accomplish. Away with such prevarication. A conscious superiority of the forceps *will*, under such circumstances, force itself upon individuals whose prejudices will ordinarily prohibit their employment. But I have gone ahead of my subject. I proposed noticing the structure and application of the Key. With regard to the shape and proportions, I think Mr. Shepherd's views correct, because the same object is attainable with the small round fulcrum as it is with one of greater dimensions; and the lacerations and contusions incident to the use of this instrument are consequently not so extensive with the one as the other. But, as I have before said, let the shape and size of the fulcrum and hook be what they may, the only attainable object is a powerful rotary motion, the gums and alveoli being the basis of the fulcrum. And unless they be sufficiently firm to sustain the quantum of force requisite to break up the attachment of the teeth to their alveolar parietes, and for their removal (in only one circumscribed direction,) a contusion or fracture is the unavoidable result, to a greater or less degree. But we are led to infer that Mr. Shepherd's Key will not slip down or rest upon the gum, but that it will remain on a line with its termination. Yet he tells us that there are some cases in which it is preferable to any instrument that he has ever seen. As he does not specify those instances, we are to infer, that he alludes to those cases in which the teeth have lost so much of their substance, on one of their sides, as to afford no hold for the beak of the forcep, and in which a hold is afforded strong enough on the other side for their removal with the key. Now, according to Mr. Shepherd's own shewing, no properly constructed key will rest upon the gums, nor will any man of *proper feeling* expose so highly sensitive a structure as the inflamed gums to the painful lacerations or contusions which he admits will result from the use of a disproportioned instrument. But where now do we find his fulcrum ? against the tooth on a line with the termination of the gum ? Oh, no—that portion of the tooth is gone ; enough is not even left to be embraced by the forceps. Where then—where can or does it rest but upon the gum and alveolar processes, where it always does ? For, adjust it as you may, in the effort to extract, the fulcrum will always encroach to a greater or less extent upon them, but in this instance the pressure is all concentrated immediately upon them. And this is an instance in which the superiority of the key is demonstrated. Another sage and philosophical deduction ! The subject here resolves itself into one proposition, whether it be not more in accordance with the principles of correct surgery, and whether it will not be contributing in a much greater degree, to the comfort of those who seek our professional aid, to remove with a sharp and well tempered instrument a small portion of the gum, together with that attenuated edge of the alveolus which is so soon taken up by the absorbents, thereby affording ample hold for a certain and safe application of the forceps, than to expose these highly sensitive parts to the pressure requisite for extraction—when too, that force is so precariously applied. That the former of these will be sustained, not only by theoretical investigation, but by practical experience, is a fact of which I entertain not a doubt. Conceiving it unnecessary to notice further this part of the subject, we will now notice the advantages of properly constructed forceps. It may not be amiss for me here to remark, that most of the forceps hitherto used were as defective as the Key, noticed by Mr. Shepherd. Their dimensions being better adapted to a smithery than to the delicate organs of the mouth. But it is with the most triumphant gratification, that, in lieu of those mis-shaped and unwieldy instruments, we can now call the attention of the profession to the conjoined production of Cartwright and Snell, and of the ingenious and accomplished Dr. Flagg. Yet important and beneficial as have been the improvements of those gentlemen, it is to be regretted, that a large number of the forceps now in use, are but illy adapted to the purposes which they should subserve. And I must here remark, that the fault lies at the operator's door—careless indifference or confidence in the capacity and disposition of the manufacturer, to turn out a perfectly finished and well adapted instrument, has perhaps resulted in greater injury both to the profession and its patrons, than most persons are aware of. Snell never uttered a more important truth, than when he said, that all operators should pay the most minute attention to the construction of their forceps, and themselves do, what no instrument maker ever would, viz : render them exactly what they should be. Most of the forceps now in use are not sufficiently hollowed out in the inner surface of their beaks, consequently a firm hold cannot be taken upon the necks of the teeth, and so much pressure is applied to their dilapidated crowns in the effort to extract, as to crush them without effecting the object desired. Properly constructed forceps should have their beaks so hollowed out by the file, as to fit the crown, and at the same time to embrace firmly the neck of the tooth. The instrument may then be applied, without the danger of crushing, and a much more effectual hold be taken. So much for the construction of the forceps—now for their application—their practical superiority. In sustaining my position, it becomes necessary for me to notice the irregularities of the shape, size, and direction which the fangs of the teeth take in their growth. By a law of the economy, the teeth have allotted for their support, a certain number of fangs, to occupy certain positions. Yet it is an every day occurrence, to witness departures from that law, in the one instance, as well as in the other; some of them being short, others long, some straight, others crooked, some converge and others as if to exhibit the caprices of nature, diverge so much as to occasionally render their safe removal, an operation of great difficulty. Then to meet and surmount the difficulties which such anomalies present, would common sense, would a knowledge of those diversities, or rather the mystery with which they are shrouded, arm us with an instrument by which these ends might be accomplished, only under favorable circumstances, with ease and safety, or would they place in our hands a variety of instruments, the construction of which would render them applicable to any and every anomaly, which might present itself! But to be more practical, let us again take a cursory glance at the application of the two instruments. The one I have partially noticed, and would only here add, that however dissimilar the circumstances, the *modus operandi* is in all instances the same. The only control the operator can exercise, is in adjusting the fulcrum on the inner and outer sides, and this advantage is of so little consequence, that it is even a controverted point which of the sides is most proper. With the other a firm and effectual hold may be taken upon any tooth which can be removed at all, without incurring the danger of either bruising or lacerating the gums, and the application of force be precisely suited to the circumstances, at the same time, lever power is afforded sufficiently great for the safe removal of any tooth. We deem it here a work of supererogation to say more; all we ask is an impartial and unprejudiced comparison of the instruments, in their most perfect state. Let their application and the result be scrutinized. That much I ask, and that is all. Lastly: Should the use of an instruments be perpetuated, when its place may be supplied by one possessing all its desirable qualities and clear of its objectionable ones? That this is practicable we think we have shewn. That it is an infinitely more painful instrument than the forceps is a fact sustained by reason, and attested by the most skilful and experienced surgeons of the present day. Why is it that we have seen evinced so much aversion to the operation of extraction? and why is it that we see this aversion gradually subsiding? The query is easily answered. The injuries resulting from the use of the Turnkey originated in a great measure the one, and its partial disuse has contributed to the other. But, as Mr. Shepherd truly observes, the objects are to remove a tooth with facility, and at the same time to avoid unnecessary pain; and he who has the ability to accomplish this object with the key ought not to exchange it for any instrument. But *who* has that ability? If any there be, the number must indeed be a limited one, for we are told by one eminent, and no doubt deservedly so, that even now (in the blaze of the nineteenth century, when the arts and sciences have attained to their greatest perfection,) few of the great number, exercising the duties of dental surgery, either know its use or understand its application. Then, I contend, that an instrument so mysterious in its use as to baffle the intelligence and skill of the generality of dentists of the present day, should be rather generally abandoned than recommended. Let the few who possess that ability use it for their own, and the benefit of the community: it is their prerogative, their duty to do it. But arm not the mass with engines of pain and send them out upon the world to perpetrate their mal-practices for the good a few may accomplish. Finally, I beg leave to say, that we are, according to the dictates of our best judgment, called upon by the interests of the profession,—by the laws of humanity,—by every principle which can actuate a philanthropic, a generous and dignified profession, to abandon so prolific a source of injury to the community and discredit to ourselves. In conclusion, I would remark, that in offering the following opinions to the profession, I have been actuated by no desire of bringing myself before the public or of getting up an unpleasant controversy with any one—my object being solely to aid in the investigation of truth. Differing with Mr. Shepherd widely as I do, for him and his opinions I entertain the most profound respect. It is my disposition to allow to him the same liberty of thought, and freedom of speech, which I think proper to exercise myself. As I have before remarked, a controversy is not sought. Yet, whenever I may deem it either due to myself, or the cause of science, I shall not be backward in giving utterance to such sentiments and opinions as I may consider fit and proper. W. W. H. Thackston.

*Farmville,* Dec. the 28th, 1840.

